# Metabolic dysfunction-associated steatotic liver disease as a systemic disorder: extrahepatic manifestations and the need for data-driven phenotyping

**DOI:** 10.3389/fendo.2026.1873215

**Published:** 2026-08-14

**Authors:** Sergejs Lobanovs, Maksims Jakovenko, Jeļena Ivanova, Jekaterina Nagaiceva, Jurijs Čižovs, Dmitrijs Bļizņuks

**Affiliations:** 1 University of Latvia, Faculty of Medicine, Riga, Latvia; 2 Department of Gastroenterology and Hepatology, Pauls Stradins Clinical University Hospital, Riga, Latvia; 3 Institute of Applied Computer Systems, Riga Technical University, Riga, Latvia; 4 Veselības Centrs 4, Riga, Latvia

**Keywords:** diabetes, extrahepatic manifestations, machine learning, MASLD, phenotyping, precision medicine

## Abstract

Metabolic dysfunction-associated steatotic liver disease (MASLD) is increasingly recognized as a systemic disorder that extends beyond hepatic steatosis to involve multiple extrahepatic organs, including the cardiovascular system, kidneys, brain, pancreas, and other organ systems. This multisystem involvement is accompanied by marked heterogeneity in clinical presentation and disease progression, supporting the concept that MASLD comprises distinct phenotypes rather than a uniform entity. Conventional classifications based solely on hepatic features are insufficient to capture this complexity, underscoring the need for phenotyping grounded in comprehensive clinical data. Published phenotyping attempts, however, differ in cohort definition, variable selection, and validation, and their subgroups are not directly comparable. Data-driven stratification frameworks use unsupervised clustering to identify latent patient subgroups characterized by distinct comorbidity profiles and disease trajectories, followed by phenotype-specific supervised modeling to refine risk prediction. Such approaches enable the identification of clinically meaningful MASLD phenotypes and support the implementation of precision medicine through personalized surveillance strategies, targeted therapeutic interventions, and prioritization of high-risk individuals. Ultimately, this paradigm improves prognostic accuracy, enhances resource allocation, and facilitates the prevention of extrahepatic complications, advancing personalized disease management in MASLD.

## 1 Introduction

Metabolic dysfunction-associated steatotic liver disease (MASLD) is the most prevalent chronic liver disease globally, affecting approximately 30-40% of adults and constituting a major component of the worldwide metabolic disease landscape (1, 2). While traditionally viewed as a liver-limited condition characterized by excessive hepatic lipid accumulation, increasing evidence indicates that MASLD is a systemic disease that contributes to dysfunction across multiple organs. Beyond progressive liver pathology - including steatohepatitis, fibrosis, cirrhosis, and hepatocellular carcinoma. MASLD is strongly associated with a wide range of extrahepatic manifestations such as cardiovascular disease (CVD), chronic kidney disease (CKD), neurodegenerative disorders, and type 2 diabetes mellitus (T2DM) (3, 4). These systemic manifestations make the clinical course of MASLD highly heterogeneous. Patients differ substantially in disease progression, severity of hepatic injury, and risk of developing extrahepatic comorbidities. While some individuals remain in relatively stable steatosis states for decades, others progress rapidly toward advanced liver disease and systemic metabolic complications.

Given this biological and clinical heterogeneity, there is an increasing need to move beyond a uniform diagnostic label toward phenotype-based characterization of MASLD. This approach is highlighted as a promising research direction in the recent European Association for the Study of the Liver (EASL), European Association for the Study of Diabetes (EASD), and European Association for the Study of Obesity (EASO) Clinical Practice Guidelines for the management of MASLD, emphasizing potential benefits of tailored risk stratification and the identification of patient subgroups with distinct susceptibility and disease trajectories (4). Phenotyping aims to identify patient subgroups with distinct metabolic, molecular, and clinical profiles that correspond to different disease mechanisms and risk trajectories. Such stratification is essential for improving prediction of disease progression and the development of extrahepatic comorbidities, enabling earlier intervention and more personalized monitoring strategies.

Recent advances in data science and machine learning provide powerful tools for uncovering latent patterns within complex clinical datasets, enabling data-driven discovery of MASLD phenotypes. Several such attempts have already been reported; as discussed in §4.1, they differ substantially in design and yield subgroups that are difficult to reconcile. By integrating laboratory biomarkers, imaging data, and longitudinal clinical outcomes, these approaches can help identify patient subgroups with shared biological characteristics and differential risk of multi-organ complications. Understanding these phenotypic structures may support precision medicine approaches in MASLD, improving risk stratification and guiding targeted prevention strategies aimed at preserving systemic metabolic health and healthspan.

## 2 Pathogenesis of metabolic dysfunction-associated steatotic liver disease

The detailed processes behind MASLD development are complex and not yet fully understood. A key factor is the excessive hepatic lipid accumulation. Previously, MASLD pathogenesis was explained by the “two-hit” model: the first hit involves an abnormal hepatic lipid accumulation due to disrupted lipid metabolism, leading to MASLD. The second hit includes liver cell damage caused by oxidative stress, inflammation, and mitochondrial dysfunction, which arises on top of this fat overload. This second hit drives the progression of MASLD to more severe states such as metabolic dysfunction-associated steatohepatitis (MASH), fibrosis, cirrhosis and hepatocellular carcinoma (5).

**Figure 1:**
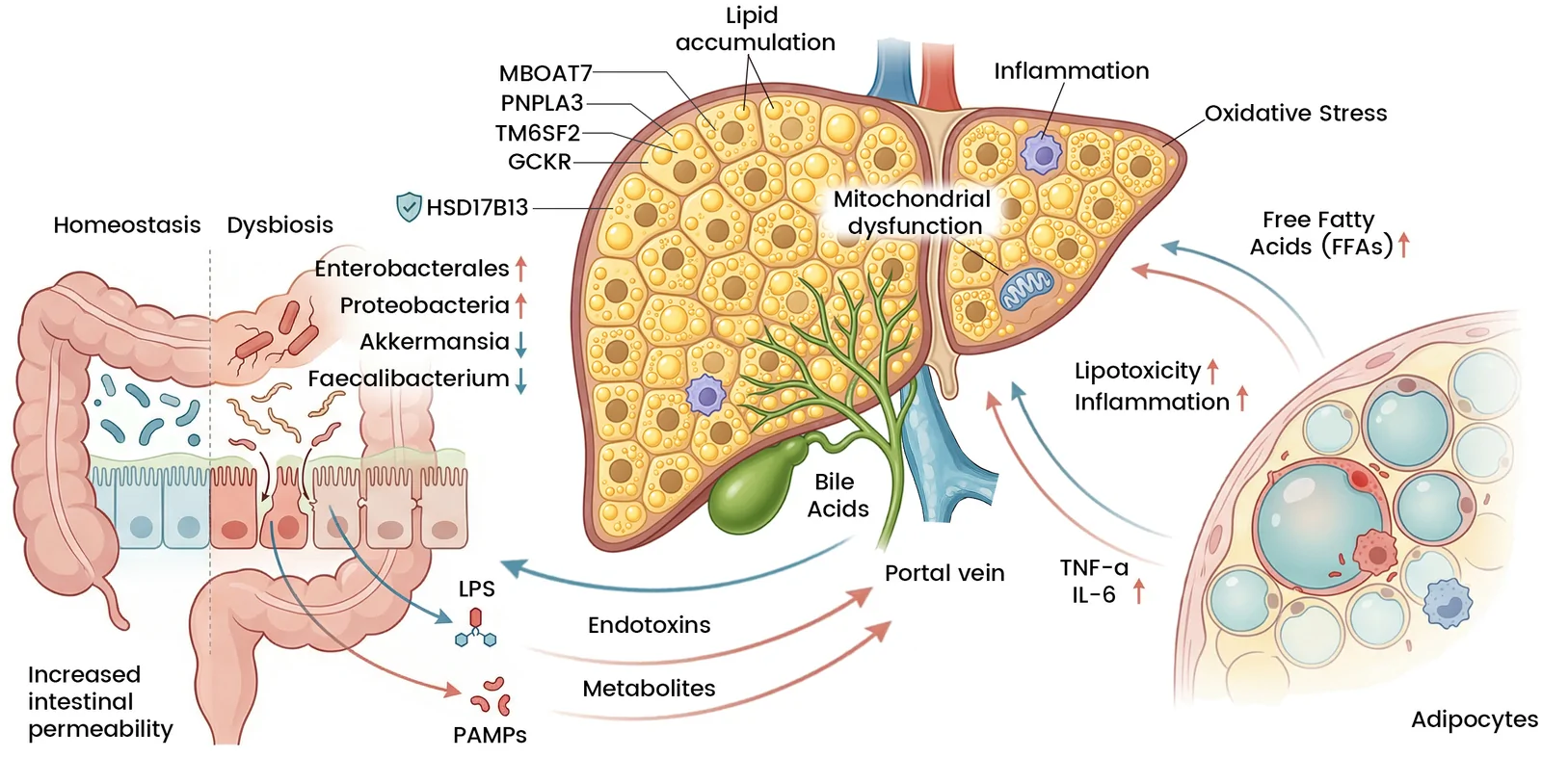
Mechanisms of metabolic dysfunction-associated steatotic liver disease development.

Recently, an improved “multiple-hit” model, complementing the previous model, has become increasingly popular. **Figure 1** presents our proposed visualization of the current model. The improved model demonstrates that interplay among hepatocytes, the gut-liver axis, and adipose tissue-representing multi-organcrosstalk-plays a pivotal role in MASLD pathogenesis. Furthermore, diverse contributors, such as *de novo* lipogenesis, lipotoxicity, insulin resistance, hyperuricemia, genetic predispositions, endoplasmic reticulum stress, drive the initiation and progression of MASLD (6, 7).

It is particularly important to emphasize the role of genetic predisposition in the development and progression of MASLD. Genetic predisposition plays a major role in MASLD pathogenesis. Genome-wide association studies have identified notable genes, including PNPLA3, TM6SF2, GCKR, MBOAT7, and HSD17B13. Notably, PNPLA3 rs738409 emerges as the principal risk variant, fostering hepatic steatosis and accelerating progression of MASLD to MASH, fibrosis, and hepatocellular carcinoma, with an associated 12-fold increased HCC risk and 18-fold increased liver-related mortality.​ MBOAT7 variants correlate with hepatic lipid accumulation and progression of hepatic inflammation (8). TM6SF2 rs58542926 disrupts very low-density lipoprotein (VLDL) secretion from hepatocytes, thereby intensifying steatosis severity, while GCKR rs1260326 promotes *de novo* lipogenesis and intrahepatic fat accumulation (9). In contrast, HSD17B13 loss-of-function alleles (e.g., rs72613567) confer protection by attenuating lipid droplet formation and mitigating MASLD susceptibility (10).

As previously mentioned, the gut microbiome plays a crucial role in MASLD development. The liver and gut are bidirectionally connected through the portal vein and bile duct (the gut-liver axis). There is growing evidence of distinct microbiota profiles across diseases, including MASLD. In patients with MASLD, these changes commonly include increased abundance of Proteobacteria and Enterobacterales, particularly Gram-negative bacteria such as Escherichia, and reduced levels of beneficial commensal species such as Akkermansia muciniphila and Faecalibacterium prausnitzii, which are important producers of anti-inflammatory metabolites like butyrate. Alterations have also been observed in the duodenal mucosal microbiome, where genera such as Serratia and Aggregatibacter appear more frequently in MASLD, suggesting that small-intestinal dysbiosis may play a significant role, especially given its connection to the liver via the bile duct system (11).

These changes in microbiota composition and function disrupt intestinal barrier integrity, reduce tight-junction proteins, and damage the mucus layer, increasing intestinal permeability (12). This disruption allows translocation of microbial products such as pathogen-associated molecular patterns (PAMPs). PAMPs like lipopolysaccharides (LPS) and microbial metabolites such as short-chain fatty acids (SCFAs), ethanol, and bile acids can move from the gut into the bloodstream and reach the liver through the portal vein. LPS activates Kupffer cells, triggering proinflammatory and fibrogenic responses. Gut dysbiosis disrupts microbial metabolite production, leading to harmful effects on hepatocytes, including inflammation that promotes fibrosis and plays an important role in MASLD pathogenesis by modulating hepatic lipid metabolism, insulin sensitivity, and inflammatory responses (13). In turn, MASLD progression alters the gut microbiome composition due to changes in bile acid metabolism and impaired bile flow, thereby reducing the delivery of IgA and antimicrobial compounds to the intestine and, as a result, favoring pathogenic overgrowth and perpetuating dysbiosis, creating a self-reinforcing vicious cycle (14).

In conclusion, the development of MASLD is driven by a complex interplay of metabolic, genetic, and environmental factors that act simultaneously rather than sequentially. The shift from the earlier “two-hit” hypothesis to a “multiple-hit” model highlights the critical role of multifaceted pathological interactions in MASLD development. Together, these mechanisms create a dynamic and self-reinforcing process that promotes disease initiation and progression, highlighting the need for a comprehensive and systemic approach to understanding MASLD pathogenesis.

## 3 Metabolic dysfunction-associated steatotic liver disease as a driver of multi-organ dysfunction

**Figure 2:**
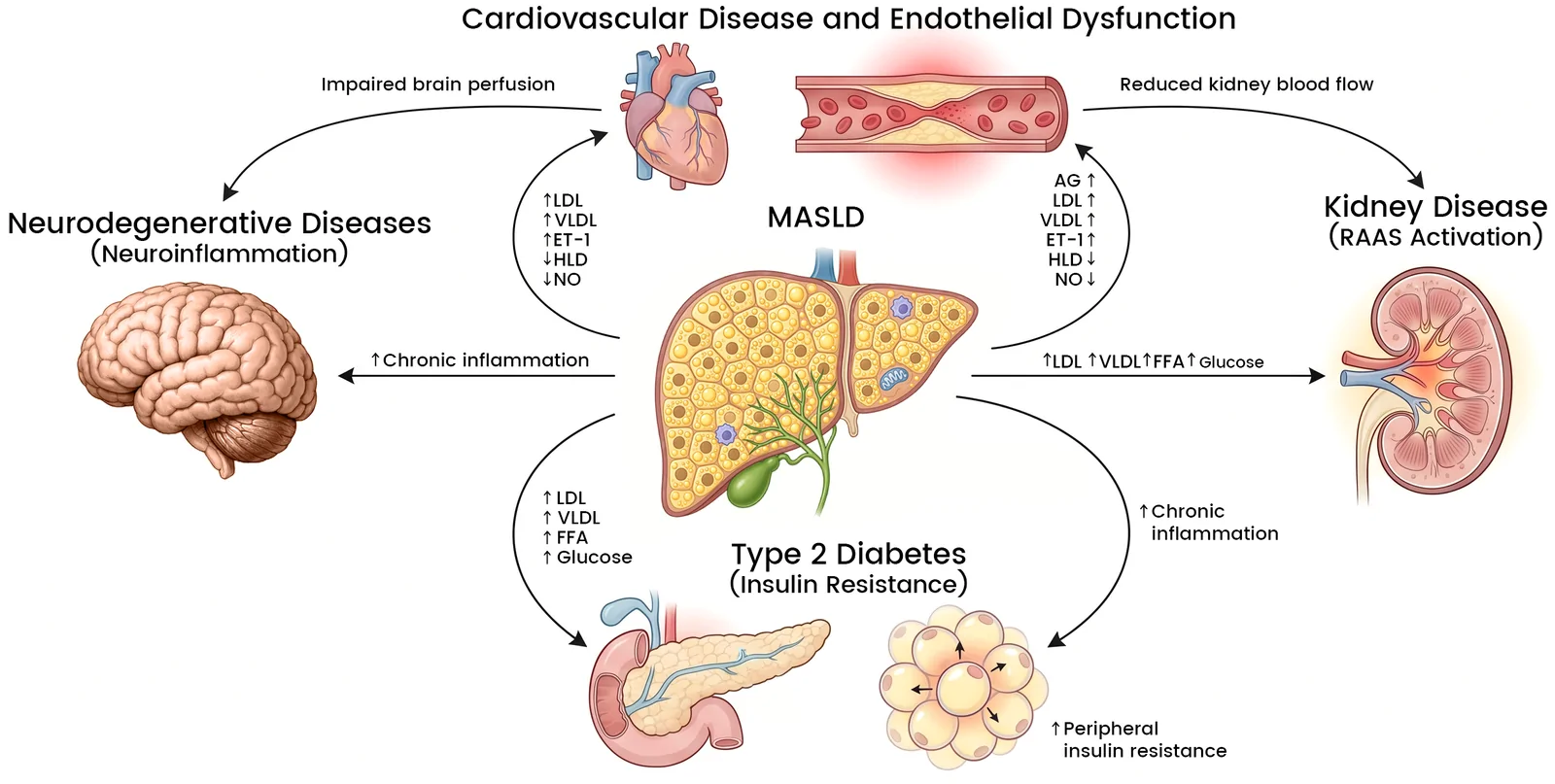
Metabolic dysfunction-associated steatotic liver disease as driver of multi organ dysfunction.

MASLD extends beyond its hepatic origin and exerts widespread effects on multiple organ systems (see **Figure 2**). Building on the complex pathophysiological mechanisms described earlier, MASLD can act as a key driver of systemic dysfunction through interconnected metabolic and inflammatory pathways. As a result, it contributes significantly to the development of extrahepatic complications, highlighting its role as a multisystem disease rather than a liver-limited condition.

### 3.1 Cardiovascular disease

MASLD is strongly associated with an increased risk of CVD and is an important contributor to cardiovascular morbidity and mortality. Individuals with MASLD have a higher incidence of major cardiovascular events, including myocardial infarction, heart failure, and cardiovascular death, compared with individuals without MASLD (15). Nationwide Korean cohort data demonstrated a higher CVD incidence among individuals with MASLD than among those without MASLD (8.5 vs. 6.2 per 1000 person-years) (16).

The vascular endothelium maintains vascular homeostasis by regulating vasodilation, thrombosis, and vascular smooth muscle cell proliferation. In MASLD, endothelial dysfunction develops due to reduced nitric oxide (NO) bioavailability, driven by oxidative stress, chronic low-grade inflammation, and increased asymmetric dimethylarginine, an inhibitor of nitric oxide synthase. Impaired NO signaling promotes vasoconstriction, hypertension, and vascular stiffness. Additionally, increased endothelin-1 (ET-1) activity contributes to vasoconstriction, vascular remodeling, and atherosclerotic progression, further increasing cardiovascular risk in MASLD (17, 18).

In MASLD, adipose tissue insulin resistance increases lipolysis and circulating free fatty acid (FFA) levels, promoting hepatic lipid accumulation and impaired insulin signaling. Enhanced hepatic lipogenesis and insulin resistance stimulate VLDL secretion, contributing to atherosclerosis characterized by elevated LDL and reduced HDL levels. These metabolic disturbances promote vascular inflammation and atherosclerotic plaque formation, thereby increasing cardiovascular disease risk (19).

Patients with MASLD, particularly those with advanced hepatic fibrosis, exhibit an increased risk of atherosclerotic progression. Although fibrosis severity demonstrates a stronger association with atherosclerosis development, MASLD itself remains an independent risk factor for cardiovascular disease progression (20).

The interplay between dysregulated lipid metabolism, insulin resistance, and chronic inflammation in MASLD contributes to atherosclerotic plaque development and increased cardiovascular risk. These metabolic abnormalities represent important pathogenic mechanisms contributing to vascular dysfunction and increased cardiovascular risk in individuals with MASLD.

### 3.2 Chronic kidney disease

Liver metabolic dysfunction has increasingly been recognized as a significant contributor to the development and progression of CKD and metabolic dysfunction-associated kidney disease (21).

The association between MASLD and CKD is well established in the epidemiological literature. A recent large-scale meta-analysis by Kueh et al. (2026) evaluated 36 observational studies, including 23 studies assessing CKD prevalence (575,615 participants) and 13 studies reporting CKD incidence (27,996 incident cases) among adults with MASLD. The pooled analysis demonstrated a global CKD prevalence of 15.2% in individuals with MASLD, indicating that approximately one in seven affected individuals also have CKD. Furthermore, the pooled incidence of CKD was estimated at 22.17 per 1000 person-years. Importantly, the coexistence of MASLD and CKD was associated with more than a two-fold increase in all-cause mortality compared with MASLD alone (18.28 versus 7.26 deaths per 1000 person-years), highlighting the substantial clinical burden associated with this comorbidity (22).

Pathologically increased hepatic lipid accumulation results in elevated production and circulation of free fatty acids and lipoproteins such as VLDL, which are taken up by the kidneys, particularly the proximal tubular epithelial cells that rely heavily on fatty acid oxidation. This ectopic lipid deposition induces lipotoxicity in kidney cells, triggering oxidative stress and inflammatory responses that promote glomerulosclerosis, tubulointerstitial fibrosis, and progressive kidney dysfunction (23).

Activation of the renin-angiotensin-aldosterone system (RAAS) contributes to the development and progression of CKD in MASLD. Inflammatory and oxidative stress-related hepatic alterations may increase angiotensinogen production, thereby promoting RAAS activation and subsequent angiotensin II generation. Angiotensin II contributes to vasoconstriction, hypertension, glomerular injury, proteinuria, and renal fibrosis, thereby accelerating kidney damage (24).

This pathophysiological interplay between the liver and kidney highlights the liver’s role as a central regulator of systemic metabolic health and underscores the importance of managing hepatic metabolic dysfunction to prevent or slow CKD progression, thereby supporting systemic healthspan preservation.

### 3.3 Neurodegenerative diseases

Recent evidence increasingly links MASLD to accelerated brain aging, cognitive decline, and an increased risk of neurodegenerative disorders through the proposed liver-brain axis (25, 26). Patients with MASLD have shown significantly increased incidence and risk of developing conditions such as Alzheimer’s disease, dementia or vascular dementia compared to those without MASLD (27, 28).​ MASLD is also associated with vascular abnormalities, including increased carotid intima-media thickness, endothelial dysfunction, arterial stiffness, and reduced cerebral blood flow, which may contribute to cerebrovascular injury (25). Furthermore, neuroimaging studies have reported reduced gray matter volume and cortical thinning in brain regions vulnerable to Alzheimer’s disease, suggesting structural alterations associated with cognitive impairment (29).

Several mechanisms have been proposed to underlie these associations, including chronic low-grade systemic inflammation, oxidative stress, and insulin resistance. Systemic inflammation may increase blood-brain barrier permeability and promote neuroinflammation through the activation of immune cells, including microglia and astrocytes (26). Insulin resistance may also disrupt cerebral insulin signaling and neuronal glucose metabolism, potentially promoting Alzheimer’s disease-related pathological processes, including impaired amyloid-beta clearance and tau abnormalities (28).

Overall, current evidence suggests that MASLD is associated with structural and functional alterations in the brain. However, longitudinal studies are needed to confirm this hypothesis, determine the direction and nature of this relationship, and clarify the biological mechanisms linking MASLD to neurodegenerative disease.

### 3.4 Type 2 diabetes

The relationship between MASLD and T2DM is bidirectional, with MASLD playing a significant role in the development and progression of T2DM. Evidence indicates that individuals with MASLD have more than twice the risk of developing T2DM over a five-year period compared to those without MASLD. This underscores the importance of recognizing MASLD not only as a liver condition but also as a critical factor in the development of systemic metabolic diseases such as T2DM (4, 30). Both MASLD and T2DM share a close association with insulin resistance.

Insulin resistance is a multifactorial metabolic disorder characterized by a diminished cellular response to insulin in key target tissues, including the liver, skeletal muscle, and adipose tissue. Among these, adipose tissue - particularly white adipose tissue (WAT) - plays a central and often initiating role in its development (31). Adipocytes possess the capacity for hypertrophic expansion to accommodate substantial lipid storage while maintaining cellular function and homeostasis. However, excessive hypertrophy triggers pro-inflammatory signaling pathways, notably involving the activation of c-Jun N-terminal kinase and IκB kinase, which contribute to the initiation and exacerbation of insulin resistance within these cells (32, 33). Increased levels of proinflammatory cytokines like tumor necrosis factor alpha and interleukin 6 are frequently observed in obese individuals with insulin resistance, indicating that inflammation contributes to the condition (33).

Impaired insulin action leads to defective suppression of lipolysis, resulting in excess release of FFA into the circulation, which contributes to insulin resistance in other tissues like muscle and liver (31). Hepatic insulin resistance reduces insulin’s ability to suppress gluconeogenesis, leading to continued glucose production. This elevates blood glucose and drives compensatory chronic hyperinsulinemia, further worsening metabolic dysfunction. It is also important to note that in obese insulin-resistant individuals, elevated circulating FFA also contribute to fasting hyperinsulinemia by directly stimulating insulin secretion (34).

Chronic hyperinsulinemia promotes hepatic insulin resistance in hepatocytes by downregulating insulin receptor expression and impairing Akt phosphorylation. *In vitro* exposure of hepatocytes to elevated insulin levels demonstrated that sustained insulin treatment markedly reduced insulin receptor levels and virtually eliminated insulin responsiveness, as evidenced by diminished Akt phosphorylation (35).

Selective hepatic insulin resistance constitutes a central mechanism underlying dysregulation of hepatic glucose and lipid metabolism. In this condition, impairment of the phosphoinositide 3-kinase-protein kinase B-forkhead box protein O1 (PI3K-Akt-FoxO1) signaling axis prevents insulin from properly suppressing gluconeogenesis. In contrast, the mechanistic target of rapamycin complex 1-sterol regulatory element-binding protein 1c (mTORC1/SREBP-1c) pathway remains relatively intact, allowing lipogenic activity to continue. This pathway-selective dysfunction generates a paradoxical metabolic phenotype characterized by concurrent hepatic glucose overproduction and unrestrained lipid synthesis.

A complementary, hepatic zonation-based framework has also been proposed to account for this phenomenon. According to this model, periportal hepatocytes become resistant to insulin’s suppression of gluconeogenesis, whereas pericentral hepatocytes remain insulin sensitive, sustaining lipogenesis. Since periportal hepatocytes are mainly responsible for gluconeogenesis, while pericentral hepatocytes specialize in glycolysis and FFA synthesis, this zone-specific difference in insulin action during hyperinsulinemia may explain why fasting hyperglycemia and hyperlipidemia occur together (36). Furthermore, circulating FFA appear to exacerbate insulin resistance preferentially within periportal hepatocytes, a phenomenon that may contribute to the heterogeneous zonal hepatocellular injury observed in metabolic liver disease (37).

All these factors contribute to the development of MASLD, and in turn, MASLD exacerbates systemic insulin resistance, thereby promoting progression to T2DM (38).

The preceding sections have demonstrated that MASLD is not merely a liver-limited disorder but a systemic metabolic disease that drives dysfunction across multiple organ systems - including the cardiovascular system, kidneys, brain and pancreas. The heterogeneity of these extrahepatic manifestations, combined with the wide interindividual variability in disease progression, underscores a fundamental clinical challenge: patients with the same MASLD diagnosis may follow markedly different trajectories of multi-organ deterioration. Addressing this challenge requires systematic, data-driven approaches capable of uncovering latent patient subgroups and stratifying risk for specific comorbidities. The following sections frame this problem within machine learning paradigms and present a structured analytical framework for MASLD phenotyping and risk stratification.

## 4 Machine learning framework for MASLD phenotyping and risk stratification

### 4.1 Existing phenotyping approaches and problem formulation

This review addresses the clinical challenge of stratifying patients with an established diagnosis of MASLD according to their underlying biological heterogeneity and differential propensity for extrahepatic disease development over time. It first examines why existing phenotyping solutions have not resolved it, and then by outline a methodological framework for doing so. Considerable interindividual variability in disease expression and progression underscores the need for systematic phenotypic characterization that can delineate patient profiles with distinct disease trajectories, improve prediction of long-term comorbidity burden, and support more personalized monitoring and preventive strategies.

In recent years, efforts have been made to characterize MASLD phenotypes using data-driven methods (see **Table 1**). A study by Gina Ryu et al., using RNA sequencing and non-negative matrix factorization, identified three distinct MASLD clusters with heterogeneous clinical and molecular profiles. Cluster I was characterized by lean patients with MASLD and relatively mild metabolic dysfunction. In contrast, Cluster III had high rates of hypertension and T2DM, along with advanced intrahepatic fibrosis. Cluster II represented obese patients with MASLD and a high prevalence of metabolic dysfunction (39). In another study, Violeta Raverdy et al. applied a data-driven clustering approach to clinical data and identified two MASLD subtypes: a liver-specific subtype associated with more rapid liver disease progression, and a cardiometabolic subtype characterized by hyperglycemia and dyslipidemia, with increased risks of CVD and T2DM (40). Another study by Chang Hong et al., using K-means clustering, identified five MASLD subtypes: metabolic-dyslipidemia, younger, obesity, inflammatory, and hepatotoxic. Metabolic outcomes differed across these subtypes; the hepatotoxic subtype showed a markedly higher risk of severe liver disease compared with the metabolic-dyslipidemia subtype, while the inflammatory subtype had the highest burden of extrahepatic complications. Accordingly, the hepatotoxic and inflammatory subtypes were classified as high-risk, and the remaining three as low-risk (41). Most recently, Huneault et al. applied unsupervised clustering to pediatric MASLD data and identified three clinically distinct metabotypes: early-mild, cardiometabolic, and inflammatory-fibrotic (42).

**Table 1:** Summary of published MASLD phenotyping studies.

Study	Population and geographic region	Sample size	Method & clustering variables	Identified phenotypes	Stability/internal validation	External validation/reproducibility	Limitations
Ryu et al. (39)	Single-center liver-biopsy cohort (healthy controls + MASLD patients); South Korea	n = 164 liver tissue samples, comprising healthy controls and MASLD patients; Clustering yielded 1 control cluster + 3 MASLD clusters	Non-negative matrix factorization-based unsupervised clustering of RNA sequencing transcriptomic data	Cluster I: lean, mild metabolic dysfunction; Cluster II: obese, marked metabolic dysfunction; Cluster III: hypertensive, diabetic, advanced fibrosis	Cluster number selected by optimal homogeneity across gene/pathway subsets; no bootstrap/resampling stability reported	None reported	The identified molecular subtypes require validation in larger, multicentre, and more diverse MASLD populations to confirm that the clustering is reproducible and generalizable across different patient groups
Raverdy et al. (40)	Bariatric-surgery cohort, France. Validation: three independent biopsy cohorts, Italy/Belgium/Finland/UK	Discovery cohort: n = 1,389 individuals with obesity undergoing bariatric surgery (histology-based diagnosis at surgery); Validation cohort: n = 1,099 MASLD patients (biopsy-confirmed) across three independent cohorts + UK: n = 6792	Partitioning-around-medoids clustering; 6 clinical variables (age, BMI, HbA1c, triglycerides, LDL-C, ALT)	Cardiometabolic and liver-specific subtypes with distinct genetic (PNPLA3, polygenic risk score) and metabolomic signatures, and differing cardiovascular/liver-event risk	Bootstrap resampling gave a mean Jaccard similarity of 0.73 (SD 0.07) across clusters, Calinski–Harabasz indices of 263 in the derivation cohort (ABOS) vs. 174 in validation, 18,774 in UK Biobank, indicating stable, well-separated clusters that transport to independent cohorts	Validated in three independent European cohorts + UV	Results depend on the six chosen clustering variables (and only the two at-risk clusters were analysed in depth), while the derivation and validation cohorts consisted almost entirely of individuals with obesity undergoing bariatric surgery, limiting generalizability to lean/overweight MASLD
Hong et al. (41)	Population-based registry, United Kingdom (UK Biobank) + hospital health-examinee cohort, China (Nanfang Hospital, Guangzhou)	n = 125,197 MASLD-diagnosed individuals (UK Biobank) + n = 995 MASLD-diagnosed individuals (Nanfang Hospital)	K-means clustering; 7 clinical indicators (age, BMI, waist-hip ratio, AST, LDL-C, cholesterol, monocyte/lymphocyte ratio)	5 subtypes: metabolic-dyslipidemia (27.5%), younger (22.7%), obesity (16.1%), inflammatory (28.8%), and hepatotoxic (4.9%)	Random 6:2:2 training/validation/internal-test split within UK Biobank; cluster-outcome associations (Cox HRs) reproduced across all three splits	External test in the Nanfang cohort confirmed cluster structure only - no outcome follow-up available	Two source cohorts differ by >100-fold in size, raising risk that cluster structure is dominated by UK Biobank
Huneault et al. (42)	Multi-site pediatric biopsy-proven MASLD cohort (NASH Clinical Research Network), United States	n = 514 children with biopsy-proven MASLD	Unsupervised clustering + metabolomics	3 metabotypes: early-mild (49.4%), cardiometabolic (36.8%), inflammatory-fibrotic (13.8%)	Not reported	Not reported	Pediatric-specific, not comparable to adult clustering studies

As observed, the methods used to address this problem rely on a range of machine learning approaches, and the identified clusters differ significantly across published studies. The four studies summarized in **Table 1** represent valuable and methodologically rigorous contributions to characterizing MASLD heterogeneity, each advancing the field through distinct and complementary analytic strategies - from transcriptomic profiling (39) to large-scale population clustering (40, 41), and pediatric metabotyping (42). However, despite these important advances, several methodological limitations constrain the comparability, reproducibility, and clinical applicability of the reported clustering solutions. A critical evaluation of these limitations is therefore essential for interpreting existing findings and guiding the development of more robust and clinically meaningful approaches to MASLD subtyping.

#### 4.1.1 Inclusion and exclusion criteria

Consistent application of current MASLD diagnostic criteria - hepatic steatosis plus at least one cardiometabolic risk factor (overweight/obesity, elevated waist circumference, dysglycaemia or type 2 diabetes, hypertriglyceridaemia, low HDL-cholesterol, or hypertension) is essential to ensure that clustered cohorts represent a homogeneous disease population rather than a mixture of related but distinct entities. Equally important is the rigorous exclusion of alternative or overlapping causes of hepatic steatosis, including significant alcohol consumption, viral hepatitis (e.g., hepatitis C), drug-induced liver injury, pregnancy-associated liver disease, and Wilson disease, among other secondary etiologies. Variability in how strictly these inclusion and exclusion criteria are applied across studies may introduce additional, unaccounted-for heterogeneity into the clustered samples, further complicating cross-study comparison and reproducibility of the resulting phenotypes (4).

#### 4.1.2 Population-specific diagnostic thresholds

Several MASLD criteria are ethnicity-dependent; waist-circumference cut-offs, for instance, differ across Europeans (≥94/≥80 cm, men/women), South Asians and Chinese (≥90/≥80 cm), and Japanese (≥85/≥90 cm). This matters most for the study by Hong et al. (41), which pools populations of different ancestry by combining a European (UK Biobank) and a Chinese (Nanfang) cohort. Applying a single uniform threshold there risks inconsistent case definition and can itself shape the resulting clusters (4).

#### 4.1.3 Sample size and cross-population validation

Adequately powered, large-scale samples are important for capturing the full spectrum of disease heterogeneity in MASLD; confirming that identified subtypes are reproducible and generalizable typically requires validation in larger, multicentre, and demographically diverse populations.

#### 4.1.4 Variable selection and model sensitivity

Cluster structure depends directly on which input variables are selected, since algorithms detect only the patterns captured by the provided parameters; a model built on six clinical markers, for instance, identifies subtypes solely along those axes, and different variable choices could plausibly yield different groupings (40). Decisions such as data scaling, handling of missing values, and the pre-specified number of clusters can likewise alter results.

#### 4.1.5 Cohort representativeness

Representation across the full body-mass-index spectrum, including lean and overweight individuals, is an important consideration for validation cohorts to ensure that identified subtypes generalize across the range of metabolic phenotypes (40).

#### 4.1.6 External validation

Even where external validation has been performed, subtype prevalence can vary appreciably across cohorts despite a broadly reproducible underlying structure (40). In other cases, external validation remains an area for future work (39, 42).

#### 4.1.7 Population specificity

Metabotypes derived from pediatric cohorts reflect a distinct developmental context and are not directly applicable to adult populations, indicating a need for age-specific validation (42).

#### 4.1.8 Cross-sectional design

Phenotype derivation across these studies relies on measurements obtained at a single time point, capturing a snapshot of a disease process that is, by nature, progressive. This may contribute to divergence in reported phenotypes across studies, since classifications anchored to a single measurement remain sensitive to the specific stage of the individual disease trajectory at which that measurement was obtained - pointing to a broader need for longitudinal studies incorporating outcome-based data over time.

Taken together, these limitations indicate that the central unresolved problem is not the absence of MASLD phenotypes, but the lack of a reproducible procedure for deriving them: existing solutions differ in cohort definition, variable selection, and validation strategy, and consequently yield clusters that cannot be compared across studies or applied to a new patient. The task can therefore be stated as follows. Given a cohort assembled under uniformly applied MASLD criteria and described by routinely available clinical and laboratory markers, patient subgroups should be derived without reference to outcomes, and accepted as phenotypes only if they show significantly different trajectories of extrahepatic comorbidity when tested against outcome data withheld from the derivation step. Such a solution is adequate only if the resulting subgroups are internally stable, reproducible in independent cohorts, and assignable prospectively to an individual patient. The present work does not report such a solution; it proposes a methodological framework for pursuing it, and specifies the analytical choices on which its validity depends.

In the following sections, we aim to clarify potential approaches to solving this problem, with a particular focus on the application of machine learning. This includes an overview of machine learning paradigms and the models commonly used in this context. Together, these strategies aim to enable data-driven phenotypic stratification of MASLD and to support precision-oriented risk assessment and management.

### 4.2 Preparing MASLD clinical data for machine learning

Real-world MASLD clinical data present several preprocessing challenges that must be addressed before applying any machine learning workflow. Missing data are common and non-random: specialized measurements such as liver stiffness and laboratory data such as insulin resistance markers (fasting insulin, Homeostatic Model Assessment for Insulin Resistance (HOMA-IR), C-peptide) or lipid metabolism markers (HDL, LDL, total cholesterol, triglycerides) are frequently absent in patients without metabolic risk flags, introducing informative missingness that simple mean imputation would distort; multiple imputation or missingness indicator encoding is preferable. High collinearity is inherent in MASLD biomarker panels - liver enzymes (ALT, AST) co-vary strongly, as do components of the lipid panel and insulin resistance proxies - making feature selection essential to prevent model instability and preserve clinical interpretability. Feature selection can be performed using model-based regularization (e.g., LASSO), wrapper methods (e.g., Boruta), or *post-hoc* importance analysis (e.g., SHAP). Finally, continuous biomarkers such as body mass index, glomerular filtration rate, and transaminases typically require normalization or log-transformation to reduce the influence of extreme values that are clinically meaningful but statistically disruptive.

### 4.3 Machine learning paradigms and analytical scenarios for phenotypic characterization of MASLD

The field of machine learning is conventionally organized into three fundamental paradigms: supervised learning, unsupervised learning, and reinforcement learning. Reinforcement learning and semi-supervised learning, while relevant in principle, require data structures (dense sequential trajectories and partially labelled cohorts, respectively) that are rarely available in current MASLD registries and are therefore beyond the scope of this review.

Thus, the design of a machine learning workflow for MASLD phenotyping is shaped by two fundamental considerations:

1. the learning paradigm - whether labelled clinical outcomes are available to guide model training (supervised learning) or whether the goal is hypothesis-free discovery of hidden patterns (unsupervised learning);
2. the data structure - whether longitudinal follow-up with time-stamped events is recorded, giving rise to censored time-to-event data, or whether outcomes are ascertained within a fixed observation window and represented as binary or categorical labels (simplified data).

**Figure 3:**
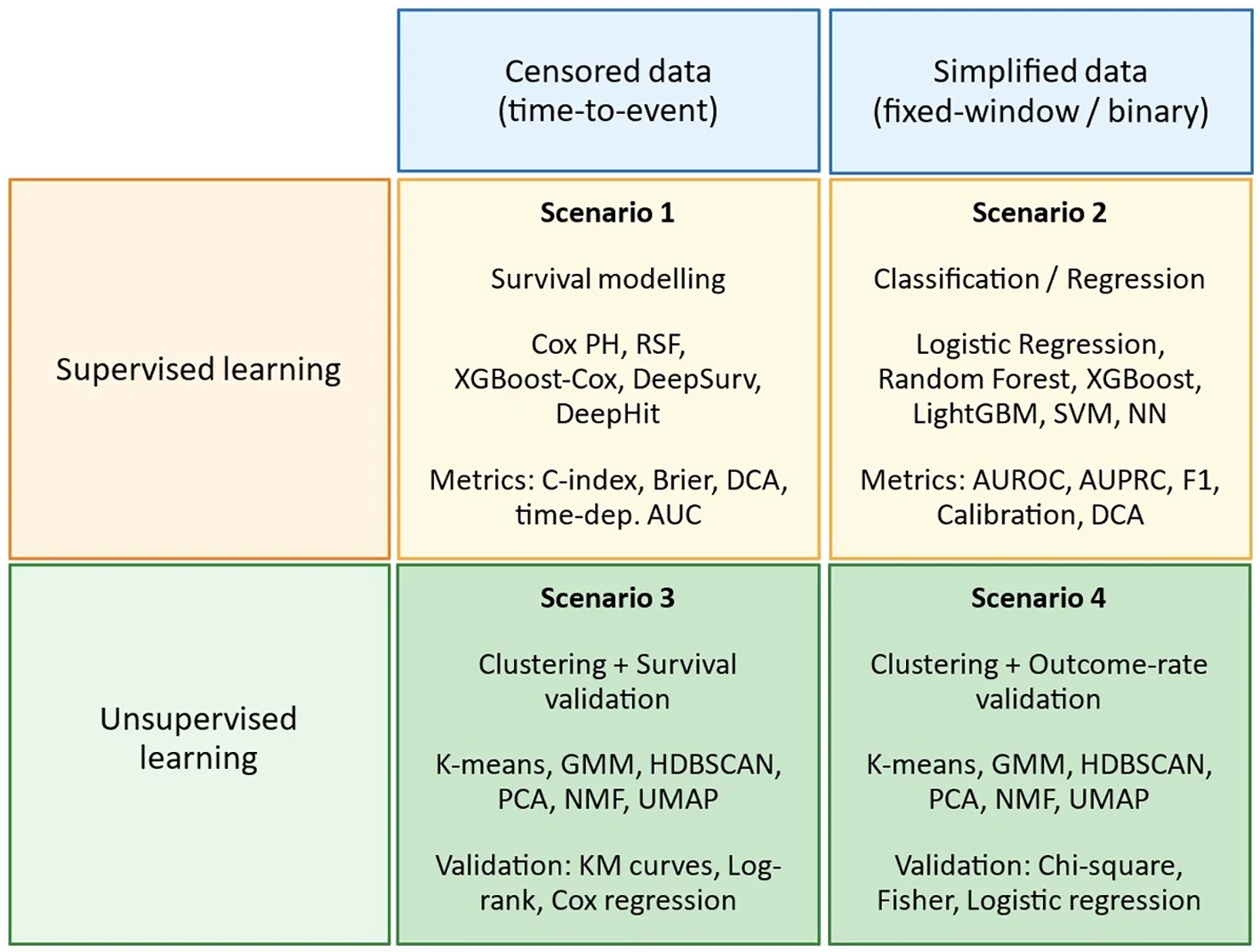
The four machine-learning scenarios for phenotypic characterization of MASLD.

Crossing these two dimensions yields four analytical scenarios (Scenarios 1–4, **Figure 3**), each with distinct methodological requirements, applicable models, and validation strategies: Scenario 1 - supervised learning on censored data (survival modelling); Scenario 2 - supervised learning on simplified data (fixed-horizon classification); Scenario 3 - unsupervised clustering with survival-based validation; Scenario 4 - unsupervised clustering with outcome-rate validation. The following subsections walk through each scenario in turn: §4.3.1 covers Scenarios 1 and 2 (the two supervised settings) and §4.3.2 covers Scenarios 3 and 4 (the two unsupervised settings).

#### 4.3.1 Supervised Learning: predicting extrahepatic comorbidity development

Supervised learning maps the biomarker feature vectors described in §4.2 to known outcome labels representing endpoints such as CVD, T2DM, CKD, or advanced fibrosis. Since MASLD patients frequently develop multiple comorbidities simultaneously or sequentially, the problem can be formulated as multi-label classification or multi-task learning when applied to an undifferentiated cohort. However, as discussed in §4.4, there are strong methodological reasons to first stratify the cohort via unsupervised phenotyping and then train supervised models within each phenotypic subgroup, where the comorbidity spectrum is narrower and the explanatory basis is already established (43).

##### 4.3.1.1 Scenario 1 - supervised learning with censored data: survival modeling

When longitudinal follow-up with time-stamped events is available, the framework extends to survival analysis, which handles censored observations and models when, not merely whether, an outcome occurs - a distinction critical for clinical decision-making, as comorbidity timing directly influences monitoring intervals and therapeutic urgency.

Applicable methods:

- Cox proportional hazards model: classical and interpretable; estimates covariate effects on the baseline hazard. Assumes proportional hazards and linear effects (44).
- Random survival forests (RSF): non-parametric; captures non-linear interactions and handles missing data natively (44, 45).
- Gradient-boosted survival models (XGBoost-Cox, LightGBM-Survival): combine gradient boosting flexibility with Cox partial likelihood loss for high-dimensional feature spaces (45).
- DeepSurv: a neural network Cox extension that captures nonlinear covariate effects; requires large cohorts (44).
- DeepHit: deep learning for competing risks; models joint event-time distributions without the proportional hazards assumption — relevant when one comorbidity alters the risk of another (44).
- Fine-Gray model: models subdistribution hazard for competing risks, enabling cumulative incidence estimation (46).

Validation: C-index, time-dependent AUC, Brier score, decision curve analysis (DCA) (44, 45, 47).

Interpretation: SHAP values, permutation importance, partial dependence plots (48, 49).

Strengths: Handles censoring; models event timing; supports competing-risk formulations essential in multi-comorbidity settings; compatible with a Markov state-transition perspective in which transitions from MASLD to comorbid states are governed by cause-specific hazards.

Limitations: Requires time-stamped events, which may be unavailable in some registries; Cox assumes proportional hazards; deep learning approaches require larger cohorts and *post-hoc* explanation.

##### 4.3.1.2 Scenario 2 - supervised learning with simplified data: fixed-horizon classification

When precise event times are unavailable or the clinical question targets a fixed horizon (e.g., “will this patient develop CVD within 2 years?”), outcomes are reduced to binary or categorical labels over a defined time window.

Applicable methods:

- Logistic regression: an interpretable baseline; penalized variants (LASSO, Ridge, Elastic Net) address biomarker collinearity and perform embedded feature selection (50).
- Support vector machines (SVM): effective for moderate-dimensional data; kernel variants capture non-linear decision boundaries (50).
- Random Forest: captures non-linear interactions, handles mixed data types, provides built-in feature importance (50).
- Gradient boosting machines (XGBoost, LightGBM, CatBoost): state-of-the-art for tabular clinical data; handle missing values natively and offer SHAP-based interpretability. In MASLD, these models have outperformed conventional scoring systems such as FIB-4 and other established fibrosis scores for the screening of advanced fibrosis (51).
- Feedforward neural networks: extend to high-dimensional spaces integrating clinical, genomic, and imaging data; require larger datasets and *post-hoc* explanation (50).

Validation: AUROC, AUPRC, F1 score, calibration curves, DCA (52).

Interpretation: SHAP values decompose each prediction into per-feature contributions (48).

Strengths: Simpler formulation; clinically familiar; works with smaller datasets; no time-stamped events required.

Limitations: Discards event timing; patients with follow-up shorter than the prediction window must be excluded or treated as negative (survivorship bias); window choice is arbitrary.

#### 4.3.2 Unsupervised learning for phenotype discovery

Unsupervised learning discovers inherent structure in data without outcome labels. For MASLD, this paradigm is especially valuable for hypothesis-free phenotype discovery - identifying patient subgroups with shared biomarker profiles that may correspond to distinct disease mechanisms and risk trajectories not anticipated by prior clinical knowledge. Dimensionality reduction is typically applied before clustering. PCA identifies linear axes of variance (often corresponding to metabolic syndrome severity, hepatic injury, or kidney decline). NMF decomposes data into additive, non-negative components that are more biologically interpretable (e.g., isolating metabolic decline, inflammatory activation, or fibrotic remodelling) and has been applied to MASLD transcriptomics (39). UMAP and t-SNE project data into 2D/3D for visual assessment of cluster separation. Autoencoders capture non-linear latent structure that linear methods may miss.

Clustering algorithms include: K-means (most commonly used in MASLD phenotyping (41); assumes spherical, equal-sized clusters); Gaussian mixture models (provide probabilistic assignments, accommodates overlapping phenotypes); hierarchical clustering (dendrograms allow *post-hoc* granularity selection); HDBSCAN (no pre-specified K, identifies outliers); and spectral clustering (detects non-convex structures via similarity graphs). Optimal K is determined through silhouette score, Davies-Bouldin index, and gap statistic, complemented by bootstrap stability analysis.

Once clusters are identified, they are characterized *post hoc* by examining the dominant biomarker domains at each centroid and validated against clinical outcomes (cardiovascular events, T2DM incidence, liver-related mortality) to confirm phenotypic relevance. The two unsupervised scenarios in our framework share the same clustering step but differ in their *post-hoc* validation strategy (43).

##### 4.3.2.1 Scenario 3 - unsupervised clustering with survival-based validation

When time-to-event data are available, cluster relevance is assessed strictly *post hoc* (outcome data are not used during clustering) via Kaplan-Meier curves with log-rank tests, multivariable Cox regression adjusting for confounders (age, sex, BMI), and competing-risks analysis (40).

Strengths: Rigorous temporal evidence of distinct risk trajectories; appropriate handling of censoring.

Limitations: Requires longitudinal follow-up; observational — causal interpretation requires caution.

##### 4.3.2.2 Scenario 4 - unsupervised clustering with outcome-rate validation

When only binary or categorical outcomes are available, validation compares prevalence across clusters via chi-square or Fisher’s exact tests, confounder-adjusted logistic regression, and outcome-distribution heatmaps (40).

Strengths: Works without event-time data; applicable to cross-sectional or limited-temporal-resolution registries.

Limitations: Less informative than survival-based validation; loses temporal granularity.

### 4.4 An integrated workflow for MASLD phenotyping

A natural question arises: if clinical outcome labels are available, why not train a supervised model directly on the entire cohort and bypass unsupervised clustering altogether? Modern gradient-boosted models can, in principle, capture patient heterogeneity internally through feature interactions. However, a purely supervised approach yields risk scores without revealing the underlying patient structure that drives those scores. The resulting model may predict well yet offer limited insight into why certain patients share similar trajectories - the very question that phenotyping seeks to answer. Unsupervised clustering addresses this by first uncovering latent patient subgroups defined by shared biomarker profiles, independently of any outcome. This discovery step serves as a hypothesis-generating phase: it identifies candidate phenotypes whose clinical relevance is then tested against held-out outcomes. Only after this validation confirms that the discovered subgroups correspond to distinct comorbidity trajectories are supervised models trained - now within phenotypically homogeneous subgroups whose biological basis is already understood. The supervised phase therefore operates on an established explanatory foundation rather than as a black-box applied to an undifferentiated cohort. This sequential approach, consisting of (i) discovering structure, (ii) confirming its clinical meaning, and (iii) predicting outcomes within the identified subgroups, motivates the integrated workflow presented below. **Table 2** summarizes how the two paradigms differ and how they combine within the workflow.

**Table 2:** Complementary use of unsupervised and supervised learning in the integrated MASLD phenotyping workflow.

Distinguishing aspects	Supervised learning	Unsupervised learning
Primary goal	Predict specific outcomes (51)	Discover latent patient subtypes (39–42)
Label requirement	Requires outcome labels (51)	No labels needed for discovery (39–42)
Clinical question	Who will develop comorbidity *X*? When will it occur (51)	What distinct patient types exist (39–42)
Output	Individual risk scores, probability estimates (51)	Cluster assignments, phenotype profiles (39–42)
Interpretability	SHAP values, feature importance (48)	Cluster characterization by dominant biomarker profiles; pathway/enrichment analysis for biological interpretation (39, 42)
Key strength	Individual risk estimates for a defined endpoint; accuracy directly measurable against known outcomes	Hypothesis-free; may reveal subtypes not captured by any single outcome variable
Key limitation	Constrained to predefined outcomes; performance degrades with low event rates or incomplete follow-up	Clinical relevance not guaranteed; clusters may reflect technical artifacts or confounders
Validation strategy	Discrimination (C-index, AUROC), calibration, clinical utility (DCA); external/prospective validation ideal (44, 45, 47)	Internal (silhouette, stability) outcome-based (survival or outcome-rate), and reproducibility in independent cohorts (39–41)
Example in clinical practice	The outcome is known in advance, for example, the presence or absence of advanced liver fibrosis. A model is trained on the clinical and laboratory data of MASLD patients with known fibrosis status and then predicts this outcome in new patients, learning the patterns associated with fibrotic progression (51)	No outcome is defined in advance. Applied to the same MASLD cohort, a clustering algorithm groups patients purely by similarity in their biomarker profiles and reveals a previously unrecognized subgroup, for example, one marked by a distinct inflammatory signature that no single fibrosis label would have isolated (39–42)
Combined unsupervised-supervised machine learning for phenotyping	First, unsupervised clustering groups patients by biomarker similarity, without reference to outcomes. Supervised models are then trained within each discovered subgroup to predict its clinical outcomes (43)	

As **Table 2** summarizes, unsupervised learning serves as the exploratory phase (hypothesis generation), while supervised learning serves as the confirmatory phase (outcome prediction within discovered subgroups). Crucially, the phenotype itself (not merely a numeric risk score) becomes a clinically interpretable deliverable: an actionable patient category that maps directly to differentiated care pathways (§4.5). A cohort-wide supervised model, however accurate, cannot provide this categorical assignment.

**Figure 4:**
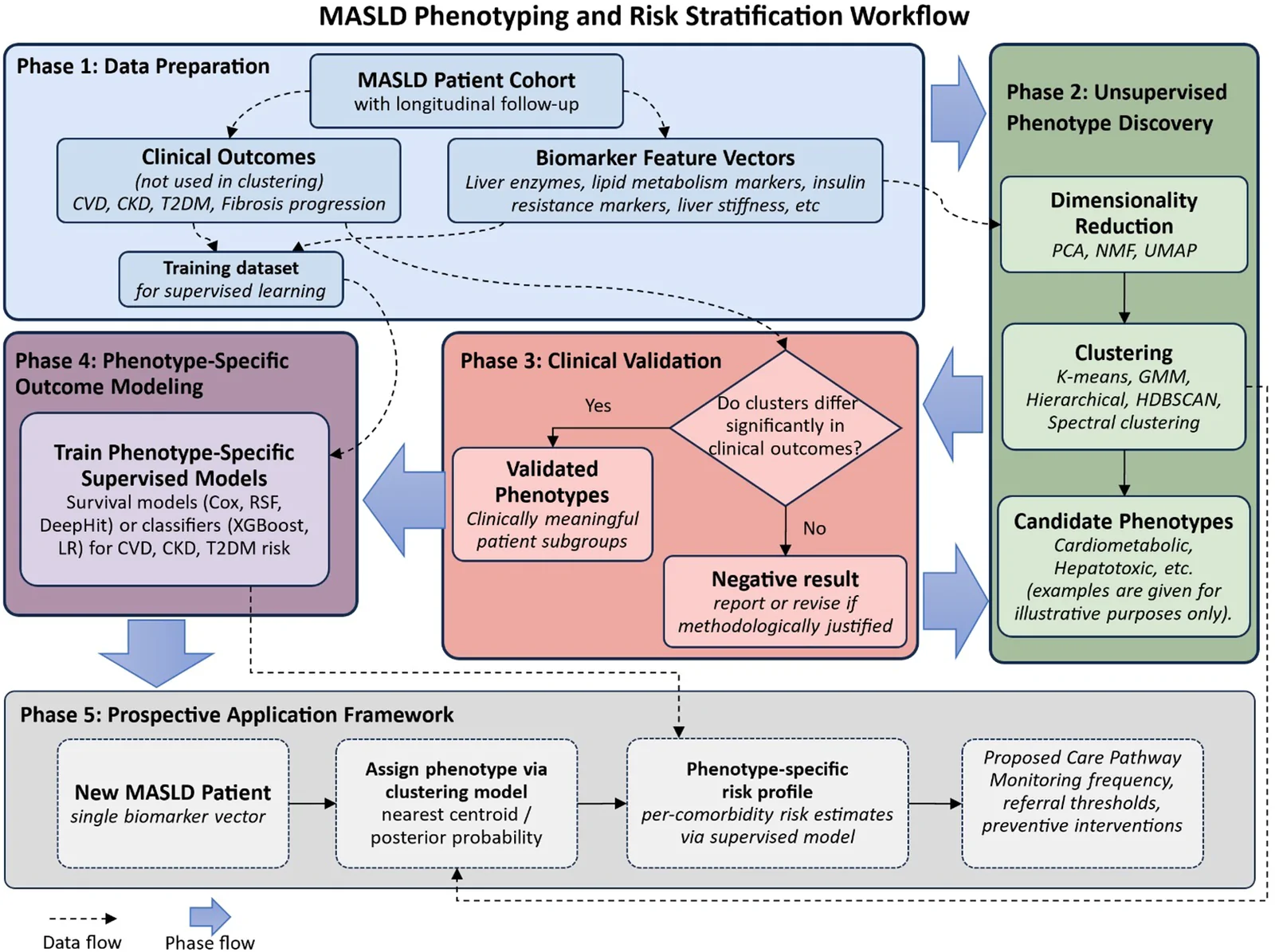
The workflow for MASLD phenotyping and risk stratification.

The resulting workflow is organized into five phases (**Figure 4**). Biomarker feature vectors are assembled during data preparation (according to §4.2) in Phase 1. Unsupervised clustering discovers candidate phenotypes without influence from outcome data (Phase 2). These candidates are validated against held-out clinical outcomes, via survival analysis (Kaplan-Meier curves, Cox regression) when time-to-event data are available (Scenario 3), or outcome-rate comparisons (chi-square tests, logistic regression) when only binary endpoints are recorded (Scenario 4 in Phase 3). Importantly, Phase 3 is an analytical checkpoint, not an optimization target. If clusters do not differ significantly in clinical outcomes, this is a legitimate finding (the biomarker space may not harbour prognostically relevant structure) and should be reported as such. Revision of the clustering setup is warranted only when independently justified (e.g., unaddressed collinearity, inappropriate cluster count, or omitted biomarker domains), not by the mere desire to achieve outcome separation, which would undermine the hypothesis-generating logic of the unsupervised step. The workflow proceeds to Phase 4 only when clusters show significantly different clinical trajectories and admit a coherent biological interpretation. Phenotype-specific supervised models, survival models (Cox, RSF, DeepHit; Scenario 1) or fixed-horizon classifiers (XGBoost, logistic regression; Scenario 2), are then trained within each subgroup to produce individualized, per-comorbidity risk scores. Phases 1–4 constitute the retrospective research pipeline. Phase 5 outlines the prospective application framework: given a new MASLD patient, the trained clustering model assigns a phenotype, the corresponding supervised model generates a per-comorbidity risk profile, and together these inform a proposed care pathway - monitoring frequency, referral thresholds, and preventive interventions tailored to the patient’s phenotypic subgroup. Phase 5 is not yet a deployed clinical system but a structured translation framework whose feasibility depends on external validation and prospective evaluation. This combined workflow forms the basis for the clinical translation discussed in the following section.

### 4.5 Illustrative worked example

To ground the workflow described above, consider a hypothetical cohort of MASLD patients that could be assembled and analyzed through the following phases.

#### 4.5.1 Phase 1 - data preparation

Exploratory data analysis would first be conducted to assess data quality, distributional properties, and the extent and patterns of missingness across key clinical and laboratory variables, as described in §4.2.

#### 4.5.2 Exclusion criteria

Participants would be excluded if they had chronic viral hepatitis, Wilson disease, pregnancy or pregnancy-associated liver disease, drug-induced liver disease, other chronic liver diseases, excessive alcohol intake, or invalid or missing liver stiffness measurements or liver biopsy data (4).

#### 4.5.3 Inclusion criteria

Inclusion would require evidence of hepatic steatosis and at least one cardiometabolic risk factor consistent with MASLD. Metabolic dysfunction was defined according to the joint EASL-EASD-EASO Clinical Practice Guidelines as the presence of overweight or obesity, increased waist circumference, dysglycaemia or type 2 diabetes, dyslipidaemia, or hypertension, including individuals receiving relevant pharmacological treatment (4).

#### 4.5.4 Phase 2 - phenotype discovery

Unsupervised clustering (e.g., K-means or Gaussian mixture modeling on a PCA- or NMF-reduced feature space, per §4.3.2), applied without reference to outcomes, might reveal MASLD phenotypes, each dominated by a distinct biomarker signature. For example, study by Chang Hong et al., using K-means clustering, identified five MASLD clusters: metabolic-dyslipidemia, younger, obesity, inflammatory, and hepatotoxic (41).

#### 4.5.5 Phase 3 - validation

These five candidate clusters would be validated against longitudinal outcome data (Scenario 3, §4.3.2). Kaplan-Meier curves and log-rank tests might demonstrate distinct prognostic trajectories across the clusters. For example, the hepatotoxic cluster could exhibit the highest risk of severe liver disease, whereas the inflammatory cluster could show the highest burden of extrahepatic complications. Such findings would support the biological and prognostic distinctness of the clusters. Groups without significant outcome separation or a coherent biomarker interpretation would be reported as such rather than forced toward significance (§4.4).

#### 4.5.6 Phase 4 - phenotype-specific prediction

If validated phenotypic clusters were identified, supervised models could subsequently be trained separately within each cluster. For example, a Cox model could be developed to predict the risk of severe liver disease within the hepatotoxic cluster, while a fixed-horizon XGBoost classifier could be applied to estimate the incidence of extrahepatic complications within the inflammatory cluster. This approach would allow each model to be optimized for the outcome profile most relevant to the specific cluster.

#### 4.5.7 Phase 5 - prospective application

A new patient entering the pipeline could first be assigned to one of the previously identified phenotypic clusters by the trained clustering model and then routed to the corresponding phenotype-specific supervised model to generate an individualized comorbidity risk profile. For example, a patient assigned to the hepatotoxic cluster could receive an estimated risk of liver fibrosis, whereas a patient assigned to the inflammatory cluster could receive a risk estimate for extrahepatic complications accordingly (§4.5).

This example remains hypothetical and illustrative, intended to demonstrate how the workflow’s phases and eligibility framework would connect in practice; the specific phenotypes, biomarker signatures, and outcome associations described here would require empirical validation in real-world MASLD cohorts.

### 4.6 From phenotype to clinical action

The objective of MASLD stratification is not classification per se, but rather translating phenotypes into clinically meaningful, individualized patterns that reflect differing disease trajectories and progression tendencies. Initial efforts toward MASLD phenotyping, as described in prior studies, demonstrate that across heterogeneous cohorts and analytical approaches, reproducible patient profiles can be identified, each corresponding to distinct pathophysiological mechanisms. This convergence provides an empirical basis for translating phenotype assignments into care pathways, and once a patient is assigned to a phenotype, that assignment should carry concrete clinical implications. For example, patient groups characterized predominantly by hepatic involvement and a propensity toward fibrotic progression require intensified hepatological monitoring and systematic fibrosis assessment (40). This has gained particular relevance with the emergence of targeted therapies such as Resmetirom, the first FDA-approved treatment for adults with non-cirrhotic steatohepatitis and moderate-to-advanced fibrosis (53). Conversely, profiles driven by cardiometabolic dysfunction necessitate regular monitoring of lipid parameters to enable timely therapeutic intervention and reduction of cardiovascular risk. Importantly, such patients also require laboratory assessment of insulin resistance to facilitate early intervention and prevent progression to T2DM (4, 40). Within this framework, computational models serve to structure rather than replace clinical judgment, linking patient-specific profiles to targeted management pathways. The clinical interventions and management strategies discussed in this work are provided for illustrative purposes only, as additional research is necessary to establish well-validated, high-confidence MASLD phenotypes.

## 5 Conclusion

MASLD extends beyond the liver, representing a systemic disorder, that underpins a wide spectrum of extrahepatic manifestations across multiple organ systems. This review highlights the marked clinical heterogeneity of MASLD and its variable trajectories of progression, emphasizing the need to reconceptualize it not as a liver-limited condition but as a multisystem disease characterized by diverse comorbidity profiles and outcomes. Such complexity necessitates a shift toward data-driven phenotyping frameworks. Stratification of MASLD heterogeneity may be achieved through unsupervised, data-driven approaches to phenotype discovery, complemented by phenotype-informed supervised predictive modelling, as outlined above. Using the previously described workflow and methodological framework, we aim to establish a foundation for clinical research aimed at elucidating MASLD phenotypes. Within this framework, the identification of clinically meaningful MASLD phenotypes represents a central objective. Advanced clustering methodologies offer the potential to delineate distinct patient subgroups with unique comorbidity patterns and disease courses, thereby refining risk stratification. This approach supports the advancement of precision medicine by enabling tailored surveillance and targeted interventions for high-risk individuals, ultimately improving prognostic accuracy and preventive strategies. Collectively, these efforts may contribute to the development of a more personalized and clinically effective management paradigm for patients with MASLD.

## References

[1] Tilg H Petta S Stefan N Targher G . Metabolic dysfunction-associated steatotic liver disease in adults: a review. Jama. (2026) 335:163–74. doi: 10.1001/jama.2025.19615 41212550

[2] Karlsen TH Sheron N Zelber-Sagi S . The EASL-Lancet Liver Commission: protecting the next generation of Europeans against liver disease complications and premature mortality. Lancet. (2022) 399:61–116. doi: 10.1016/s0140-6736(21)01701-3 34863359

[3] Bai W Zhu Z . Multiorgan crosstalk in MASLD/MASH: from hepatic pathogenesis to systemic complications. Front Endocrinol (Lausanne). (2025) 16:1720780. doi: 10.3389/fendo.2025.1720780 41488134 PMC12756970

[4] Tacke F Horn P Wai-Sun Wong V . EASL-EASD-EASO clinical practice guidelines on the management of metabolic dysfunction-associated steatotic liver disease (MASLD). J Hepatol. (2024) 81:492–542. doi: 10.1016/j.jhep.2024.04.031 38851997

[5] He QJ Li YF Zhao LT Lin CT Yu CY Wang D . Recent advances in age-related metabolic dysfunction-associated steatotic liver disease. World J Gastroenterol. (2024) 30:652–62. doi: 10.3748/wjg.v30.i7.652 38515956 PMC10950625

[6] Yang Z Zhao J Xie K Tang C Gan C Gao J . MASLD development: from molecular pathogenesis toward therapeutic strategies. Chin Med J (Engl). (2025) 138:1807–24. doi: 10.1097/cm9.0000000000003629 40640088 PMC12321477

[7] Chang Z Liu Z . Serum uric acid as a biomarker for metabolic dysfunction-associated steatotic liver disease: insights from ultrasound elastography in a Chinese cohort. BMC Gastroenterol. (2025) 25:94. doi: 10.1186/s12876-025-03666-9 39979872 PMC11841239

[8] Kumar G Shah YR Shahzad A Jameel K Guevara-Lazo D Khan NA . Genetic predeterminants and recent advancements in steatotic liver disease: a roadmap toward precision hepatology. World J Hepatol. (2025) 17:111576. doi: 10.4254/wjh.v17.i11.111576 41368108 PMC12683380

[9] Saliba-Gustafsson P Härdfeldt J Pedrelli M Parini P . Genomic signatures of MASLD: how genomics is redefining our understanding of metabolic liver disease. Int J Mol Sci. (2025) 26:10881. doi: 10.3390/ijms262210881 41303369 PMC12652521

[10] Sookoian S Rotman Y Valenti L . Genetics of metabolic dysfunction-associated steatotic liver disease: the state of the art update. Clin Gastroenterol Hepatol. (2024) 22:2177–2187.e3. doi: 10.1016/j.cgh.2024.05.052 39094912 PMC11512675

[11] Dua A Kumari R Singh M Kumar R Pradeep S Ojesina AI . Metabolic dysfunction-associated steatotic liver disease (MASLD): the interplay of gut microbiome, insulin resistance, and diabetes. Front Med (Lausanne). (2025) 12:1618275. doi: 10.3389/fmed.2025.1618275 40893881 PMC12392282

[12] Zerem E Kunosic S Kurtcehajic A Zerem D Zerem O . Bile acids in metabolic dysfunction-associated steatotic liver disease. World J Hepatol. (2025) 17:108606. doi: 10.4254/wjh.v17.i8.108606 40901599 PMC12400426

[13] Schnabl B Damman CJ Carr RM . Metabolic dysfunction-associated steatotic liver disease and the gut microbiome: pathogenic insights and therapeutic innovations. J Clin Invest. (2025) 135:e186423. doi: 10.1172/jci186423 40166938 PMC11957707

[14] Zhu F Zheng S Zhao M Shi F Zheng L Wang H . The regulatory role of bile acid microbiota in the progression of liver cirrhosis. Front Pharmacol. (2023) 14:1214685. doi: 10.3389/fphar.2023.1214685 37416060 PMC10320161

[15] Chung GE Yu SJ Yoo JJ Cho Y Lee KN Shin DW . Metabolic dysfunction-associated steatotic liver disease increases cardiovascular disease risk in young adults. Sci Rep. (2025) 15:5777. doi: 10.1038/s41598-025-89293-6 39962282 PMC11832902

[16] Moon JH Jeong S Jang H Koo BK Kim W . Metabolic dysfunction-associated steatotic liver disease increases the risk of incident cardiovascular disease: a nationwide cohort study. EClinicalMedicine. (2023) 65:102292. doi: 10.1016/j.eclinm.2023.102292 37954905 PMC10632413

[17] Zheng H Sechi LA Navarese EP Casu G Vidili G . Metabolic dysfunction-associated steatotic liver disease and cardiovascular risk: a comprehensive review. Cardiovasc Diabetol. (2024) 23:346. doi: 10.1186/s12933-024-02434-5 39342178 PMC11439309

[18] Branković M Dukić M Gmizić T Popadić V Nikolić N Sekulić A . New therapeutic approaches for the treatment of patients with metabolic dysfunction-associated steatotic liver disease (MASLD) and increased cardiovascular risk. Diagnostics (Basel). (2024) 14:229. doi: 10.3390/diagnostics14020229 38275476 PMC10814440

[19] Mellemkjær A Kjær MB Haldrup D Grønbæk H Thomsen KL . Management of cardiovascular risk in patients with metabolic dysfunction-associated steatotic liver disease. Eur J Intern Med. (2024) 122:28–34. doi: 10.1016/j.ejim.2023.11.012 38008609

[20] Lee HH Cho Y Choi YJ Huh BW Lee BW Kang ES . Non-alcoholic steatohepatitis and progression of carotid atherosclerosis in patients with type 2 diabetes: a Korean cohort study. Cardiovasc Diabetol. (2020) 19:81. doi: 10.1186/s12933-020-01064-x 32534588 PMC7293796

[21] Bansal A Chonchol M . Metabolic dysfunction-associated kidney disease: pathogenesis and clinical manifestations. Kidney Int. (2025) 108:194–200. doi: 10.1016/j.kint.2025.01.044 40379048

[22] Kueh MTW Yeo JKW Chen Y Chua NYZ Koh HL Khaw SP . Global prevalence, incidence, and outcomes of coexisting MASLD and chronic kidney disease: a meta-analysis. Liver Int. (2026) 46:e70747. doi: 10.1111/liv.70747 42347761

[23] Rong J Zhang Z Peng X Li P Zhao T Zhong Y . Mechanisms of hepatic and renal injury in lipid metabolism disorders in metabolic syndrome. Int J Biol Sci. (2024) 20:4783–98. doi: 10.7150/ijbs.100394 39309427 PMC11414397

[24] Bai J Zhang L He L Zhou Y . Altered mitochondrial function: a clue therapeutic strategies between metabolic dysfunction-associated steatotic liver disease and chronic kidney disease? Front Nutr. (2025) 12:1613640. doi: 10.3389/fnut.2025.1613640 40584092 PMC12202597

[25] Mikkelsen ACD Kjærgaard K Schapira AHV Mookerjee RP Thomsen KL . The liver-brain axis in metabolic dysfunction-associated steatotic liver disease. Lancet Gastroenterol Hepatol. (2025) 10:248–58. doi: 10.1016/s2468-1253(24)00320-0 39701123

[26] Wang J Yang R Miao Y Zhang X Paillard-Borg S Fang Z . Metabolic dysfunction-associated steatotic liver disease is associated with accelerated brain ageing: a population-based study. Liver Int. (2025) 45:e70109. doi: 10.1111/liv.70109 40296771 PMC12038381

[27] Shin WY Kang ES Oh YH Sha M Xia Q Jeong S . Metabolic dysfunction-associated steatotic liver disease, metabolic alcohol-related liver disease, and incident dementia: a nationwide cohort study : MASLD, MetALD, and dementia risk. BMC Gastroenterol. (2025) 25:308. doi: 10.1186/s12876-025-03814-1 40301749 PMC12039214

[28] Zhang J Wang W Hou X Wu J Wang Y Fan J . Metabolic-associated steatotic liver disease and risk of Alzheimer's disease: a real-world retrospective cohort study. Front Endocrinol (Lausanne). (2024) 15:1451908. doi: 10.3389/fendo.2024.1451908 39296714 PMC11408170

[29] Fan W Yang S Wei Y Tian M Liu Q Li X . Characterization of brain morphology associated with metabolic dysfunction-associated steatotic liver disease in the UK Biobank. Diabetes Obes Metab. (2025) 27:3419–30. doi: 10.1111/dom.16362 40171859

[30] Goto H Takamura T . Metabolic dysfunction-associated steatotic liver disease complicated by diabetes: pathophysiology and emerging therapies. J Obes Metab Syndr. (2025) 34:224–38. doi: 10.7570/jomes25017 40537153 PMC12318698

[31] Handy RM Holloway GP . Insights into the development of insulin resistance: unraveling the interaction of physical inactivity, lipid metabolism and mitochondrial biology. Front Physiol. (2023) 14:1151389. doi: 10.3389/fphys.2023.1151389 37153211 PMC10157178

[32] Dhokte S Czaja K . Visceral adipose tissue: the hidden culprit for type 2 diabetes. Nutrients. (2024) 16:1015. doi: 10.3390/nu16071015 38613048 PMC11013274

[33] Elkanawati RY Sumiwi SA Levita J . Impact of lipids on insulin resistance: insights from human and animal studies. Drug Des Devel Ther. (2024) 18:3337–60. doi: 10.2147/dddt.s468147 39100221 PMC11298177

[34] Fryk E Olausson J Mossberg K Strindberg L Schmelz M Brogren H . Hyperinsulinemia and insulin resistance in the obese may develop as part of a homeostatic response to elevated free fatty acids: a mechanistic case-control and a population-based cohort study. EBioMedicine. (2021) 65:103264. doi: 10.1016/j.ebiom.2021.103264 33712379 PMC7992078

[35] Bonnet L Alexandersson I Baboota RK Kroon T Oscarsson J Smith U . Cellular senescence in hepatocytes contributes to metabolic disturbances in NASH. Front Endocrinol (Lausanne). (2022) 13:957616. doi: 10.3389/fendo.2022.957616 36072934 PMC9441597

[36] He B Copps KD Stöhr O Liu B Hu S Joshi S . Spatial regulation of glucose and lipid metabolism by hepatic insulin signaling. Cell Metab. (2025) 37:1568–1583.e7. doi: 10.1016/j.cmet.2025.03.015 40245868 PMC12221773

[37] Truong XT Lee DH . Hepatic insulin resistance and steatosis in metabolic dysfunction-associated steatotic liver disease: new insights into mechanisms and clinical implications. Diabetes Metab J. (2025) 49:964–86. doi: 10.4093/dmj.2025.0644 40935652 PMC12436041

[38] Williams DM Ali J Cragg J Ch'ng CL Williams NW Stephens JW . The bidirectional relationship between type 2 diabetes and metabolic dysfunction-associated steatotic liver disease: a retrospective cohort study. Cureus. (2024) 16:e75993. doi: 10.7759/cureus.75993 39835079 PMC11743228

[39] Ryu G Yoon EL Kim W Jun DW . Molecular clustering of metabolic dysfunction-associated steatotic liver disease based on transcriptome analysis. Diagnostics (Basel). (2025) 15:342. doi: 10.3390/diagnostics15030342 39941272 PMC11817575

[40] Raverdy V Tavaglione F Chatelain E Lassailly G De Vincentis A Vespasiani-Gentilucci U . Data-driven cluster analysis identifies distinct types of metabolic dysfunction-associated steatotic liver disease. Nat Med. (2024) 30:3624–33. doi: 10.1038/s41591-024-03283-1 39653777 PMC11645276

[41] Hong C Liang SX Li ZY Li RN Zhu HB He M . Novel subtypes of metabolic associated steatotic liver disease linked to clinical outcomes: implications for precision medicine. J Transl Med. (2025) 23:769. doi: 10.1186/s12967-025-06670-5 40640848 PMC12247398

[42] Huneault HE Tiwari P Jarrell ZR Smith MR Chen C-Y Ramirez Tovar A . Clinically distinct metabotypes of pediatric MASLD identified through unsupervised clustering of NASH CRN data. Nat Commun. (2026) 17(1):3107. doi: 10.1038/s41467-026-69735-z 41735280 PMC13039967

[43] Ma EY Kim JW Lee Y Cho SW Kim H Kim JK . Combined unsupervised-supervised machine learning for phenotyping complex diseases with its application to obstructive sleep apnea. Sci Rep. (2021) 11:4457. doi: 10.1038/s41598-021-84003-4 33627761 PMC7904925

[44] Obite CP Chukwudi EO Uchechukwu M Nwosu UI . Factor enhanced DeepSurv: a deep learning approach for predicting survival probabilities in cirrhosis data. Comput Biol Med. (2025) 189:109963. doi: 10.1016/j.compbiomed.2025.109963 40037171

[45] Rhaiem R Abdo A Calderaro J Dokmak S Herrero A Toubert C . Machine learning models for disease-free survival analysis after liver resection for hepatocellular carcinoma: a multicentric French collaborative study. Liver Cancer. (2026). doi: 10.1159/000550554 41868609 PMC13004632

[46] Zhang X Zhang MJ Fine J . A proportional hazards regression model for the subdistribution with right-censored and left-truncated competing risks data. Stat Med. (2011) 30:1933–51. doi: 10.1002/sim.4264 21557288 PMC3408877

[47] Cai W Qi Y Zheng L Wu H Yang C Zhang R . Comparison of random survival forest based-overall survival with deep learning and Cox proportional hazard models in HER-2-positive HR-negative breast cancer. Cancer Rep (Hoboken). (2025) 8:e70262. doi: 10.1002/cnr2.70262 40624807 PMC12234386

[48] Almusallam N Khan S . Chronic liver disease classification using deep learning with SHAP-optimized hybrid features. iScience. (2025) 28:113972. doi: 10.1016/j.isci.2025.113972 41362625 PMC12682044

[49] Evans MD Diaz J Adamusiak AM Pruett TL Kirchner VA Kandaswamy R . Predictors of survival after liver transplantation in patients with the highest acuity (MELD ≥40). Ann Surg. (2020) 272:458–66. doi: 10.1097/sla.0000000000004211 32740239 PMC7855276

[50] Song J Ma N Aini R Yang Y . A machine learning model for non-invasive prediction of advanced liver fibrosis in patients with chronic hepatitis B. Am J Transl Res. (2025) 17:4939–51. doi: 10.62347/kevq8263 40821076 PMC12351616

[51] Dabbah S Mishani I Davidov Y Ben Ari Z . Implementation of machine learning algorithms to screen for advanced liver fibrosis in metabolic dysfunction-associated steatotic liver disease: An in-depth explanatory analysis. Digestion. (2025) 106:189–202. doi: 10.1159/000542241 39462487 PMC12129435

[52] Song K Kwon YJ Lee E Youn YH Baik SJ Lee HS . Machine learning-based model for predicting metabolic dysfunction-associated steatotic liver disease using non-invasive parameters in young adults. Front Endocrinol (Lausanne). (2025) 16:1701729. doi: 10.3389/fendo.2025.1701729 41476919 PMC12747903

[53] Tiwari A Sharma A Kumar H Gupta V Deshpande V Mupparaju JS . Resmetirom for MASH: A comprehensive review of a novel therapeutic frontier. Biomedicines. (2025) 13:2079. doi: 10.3390/biomedicines13092079 41007643 PMC12467964

